# Supplementary material for: Deciphering Reserve Mobilization, Antioxidant Potential, and Expression Analysis of Starch Synthesis in Sorghum Seedlings under Salt Stress
Source: Plants (Basel). 2021 Nov 15;10(11):2463. doi: 10.3390/plants10112463 (PMC8623787; doi:10.3390/plants10112463)
Supplement: Supplementary file 1 [file plants-10-02463-s001.zip › plants-1429008-supplementary.pdf]

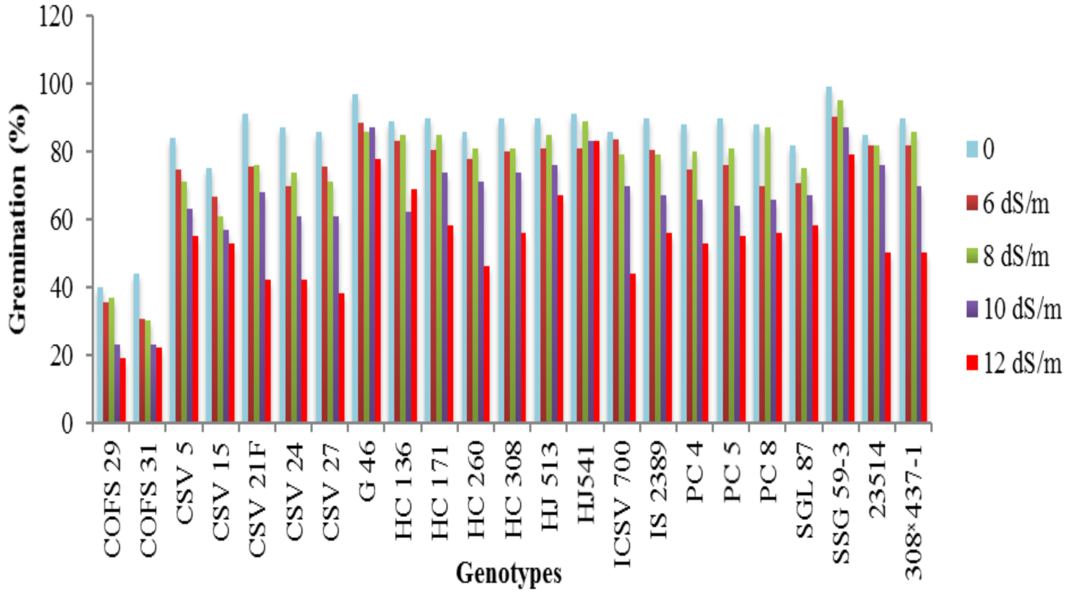

Figure S1. Effect of salt stress on germination (%) in sorghum genotypes

C.D. 1.887 **SE(d)** 0.958 **SE(m)** 1.547

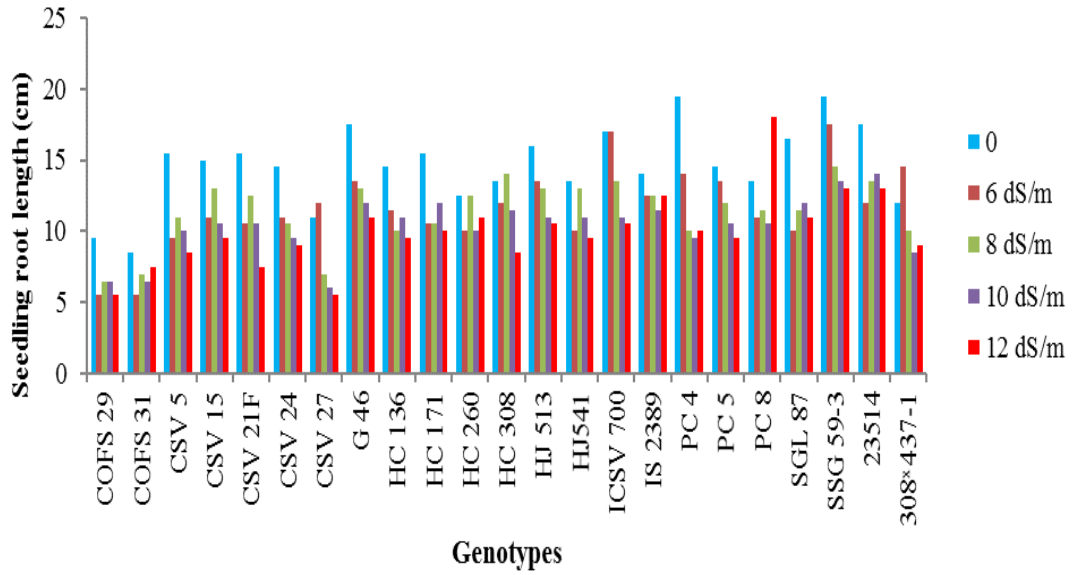

Figure S2. Effect of salt stress on seedling root length (cm) in sorghum genotypes

C.D. 0.689 **SE(d)** 0.350 **SE(m)** 0.247

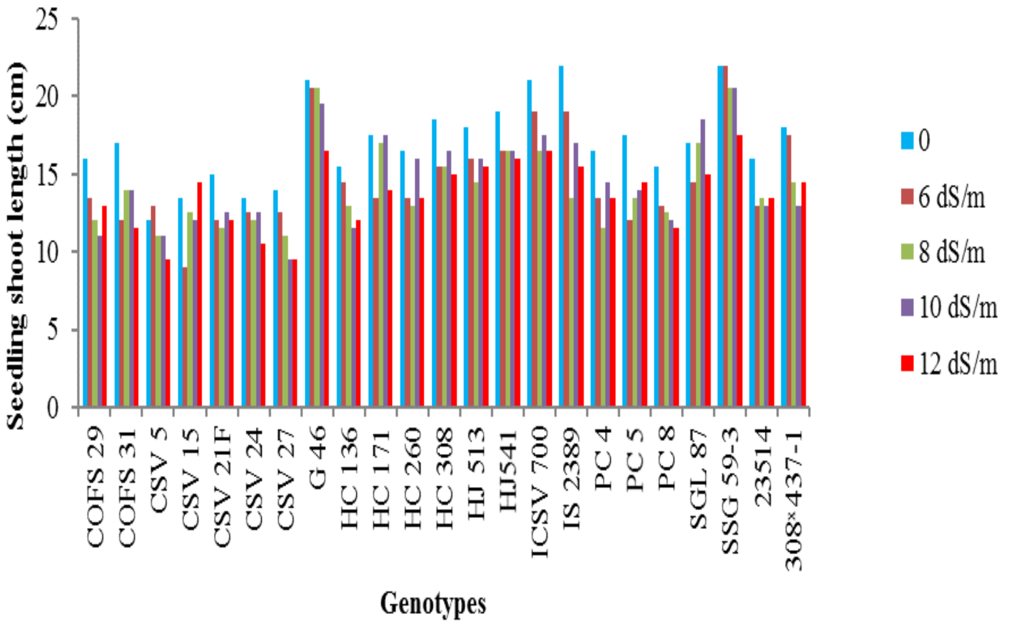

Figure S3. Effect of salt stress on seedling shoot length (cm) in sorghum genotypes

C.D. 0.646 **SE(d)** 0.328 **SE(m)** 0.232

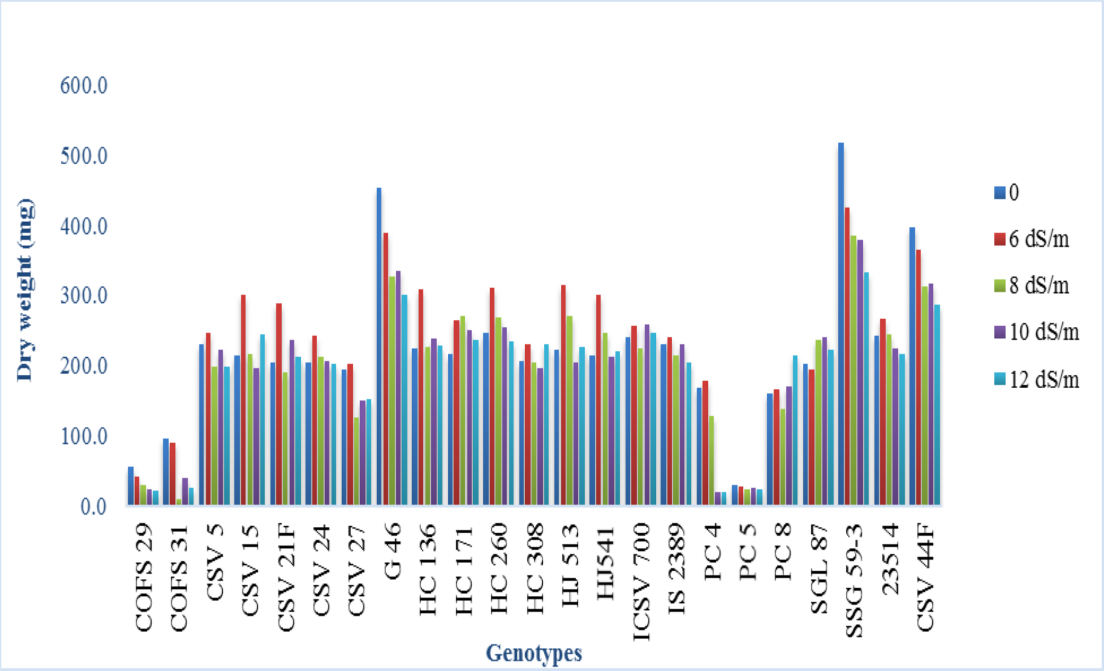

Figure S4. Effect of salt stress on dry seedling weight (mg) in sorghum genotypes

C.D. 1.546 **SE(d)** 0.623 **SE(m)** 0.546

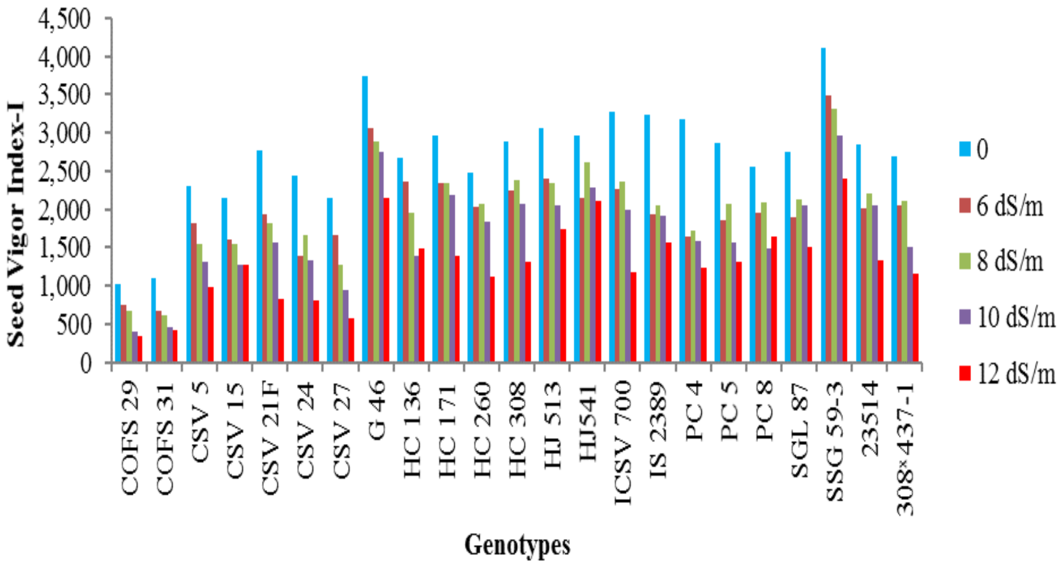

Figure S5. Effect of salt stress on vigor index-I in sorghum genotypes

C.D. 81.311 **SE(d)** 41.280 **SE(m)** 29.190

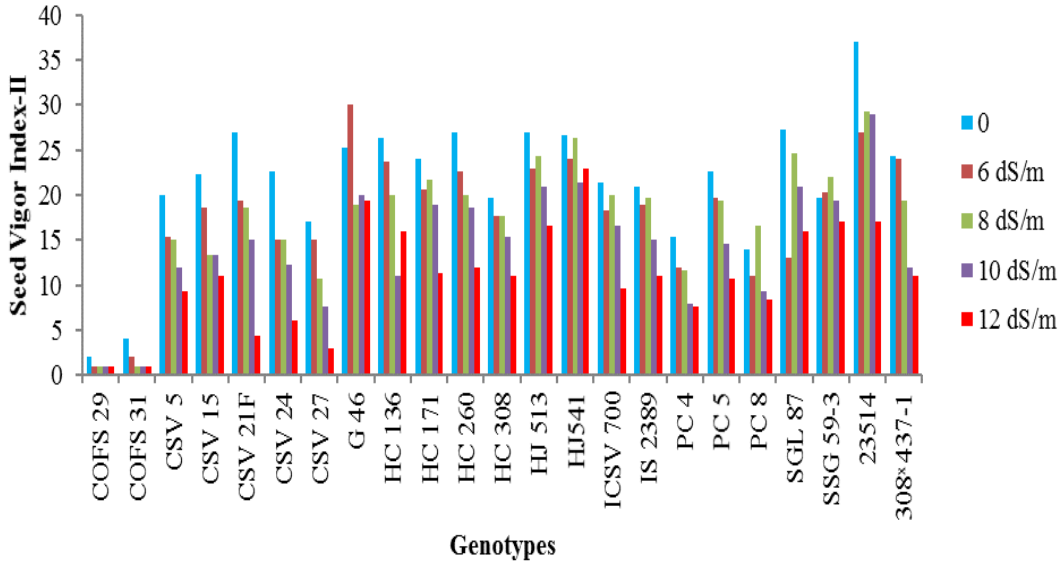

Figure S.6. Effect of salt stress on vigor index-II in sorghum genotypes

C.D. 0.838 **SE(d)** 0.426 **SE(m)** 0.301

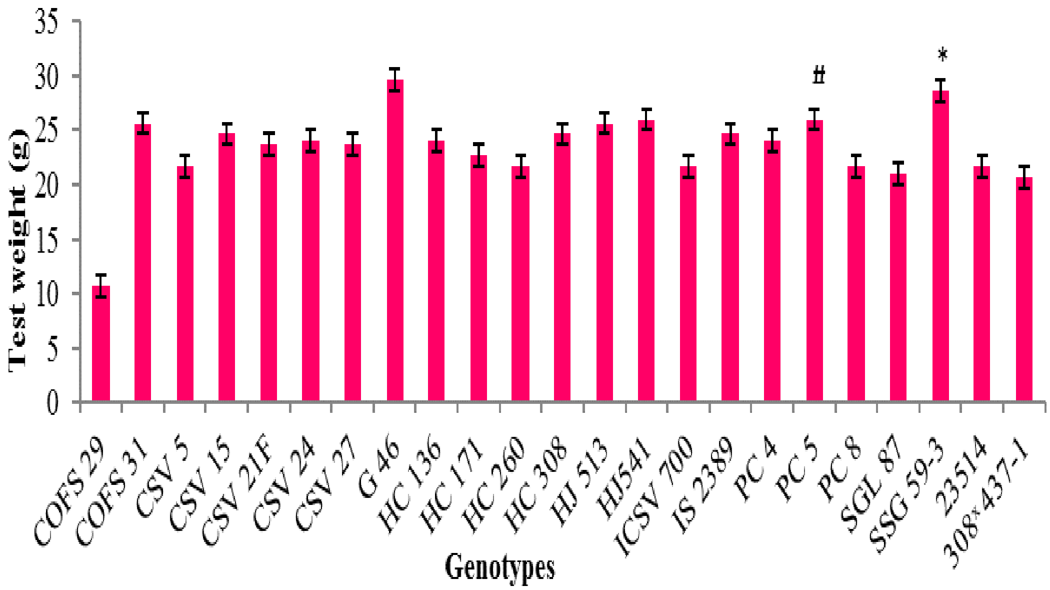

Figure S7. Test weight (g) in sorghum genotypes

C.D. 1.808

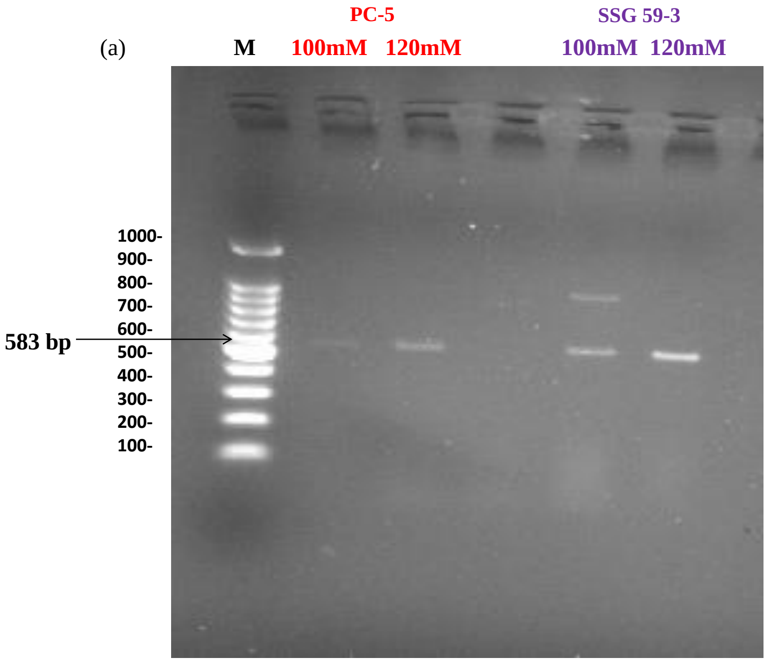

(b)

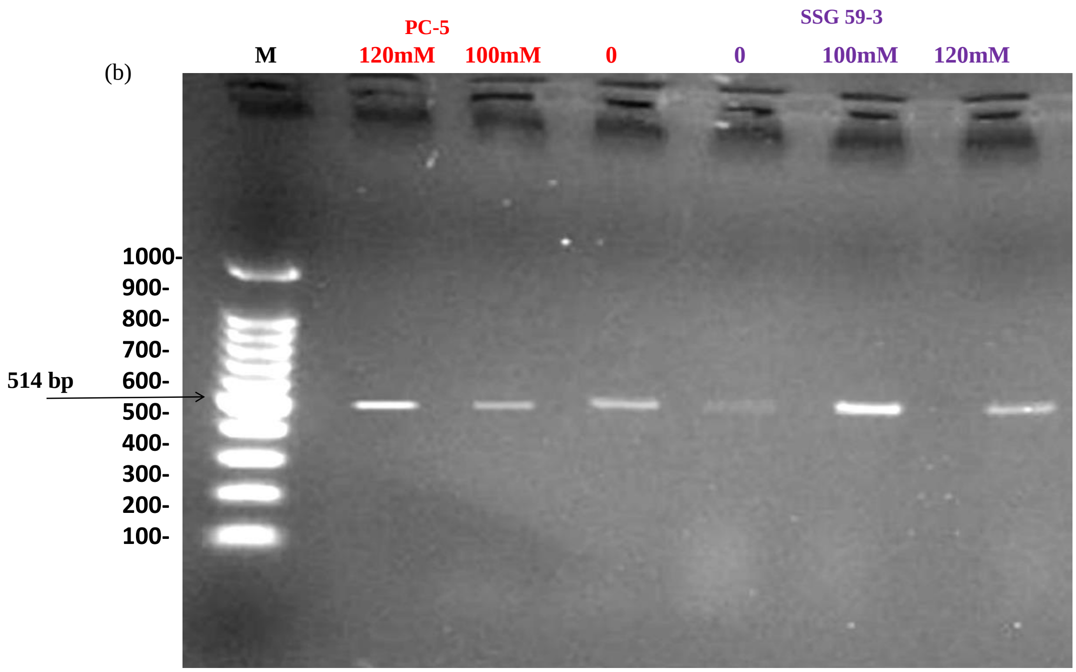
PC-5
SSG 59-3
M
120mM
100mM
0
0
100mM
120mM
1000-
900-
800-
700-
600-
500-
400-
300-
200-
100-
514 bp

(c)

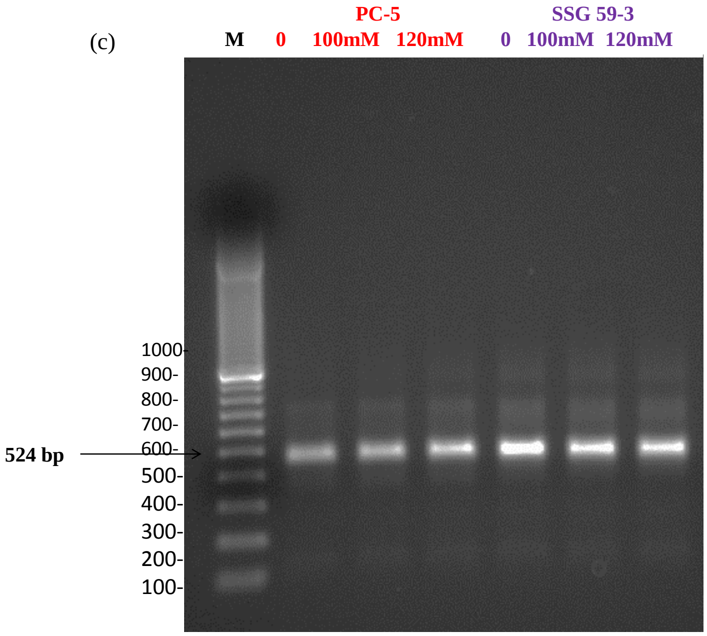
PC-5
SSG 59-3
M
0
100mM
120mM
0
100mM
120mM
1000-
900-
800-
700-
600-
500-
400-
300-
200-
100-
524 bp

(d)

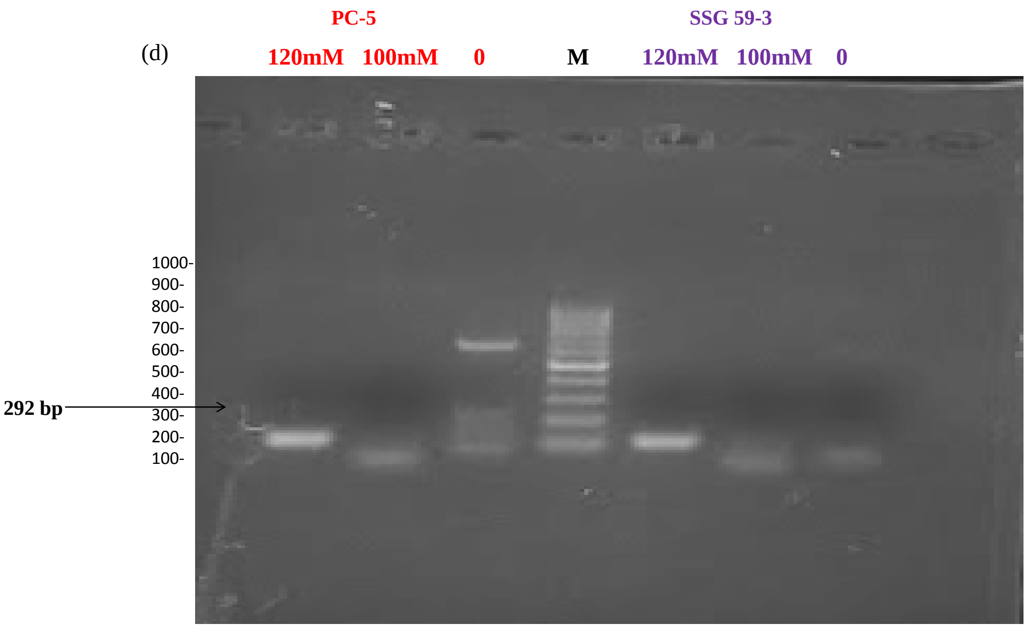
PC-5
120mM 100mM 0
M
SSG 59-3
120mM 100mM 0
1000-
900-
800-
700-
600-
500-
400-
300-
200-
100-
292 bp

(e)

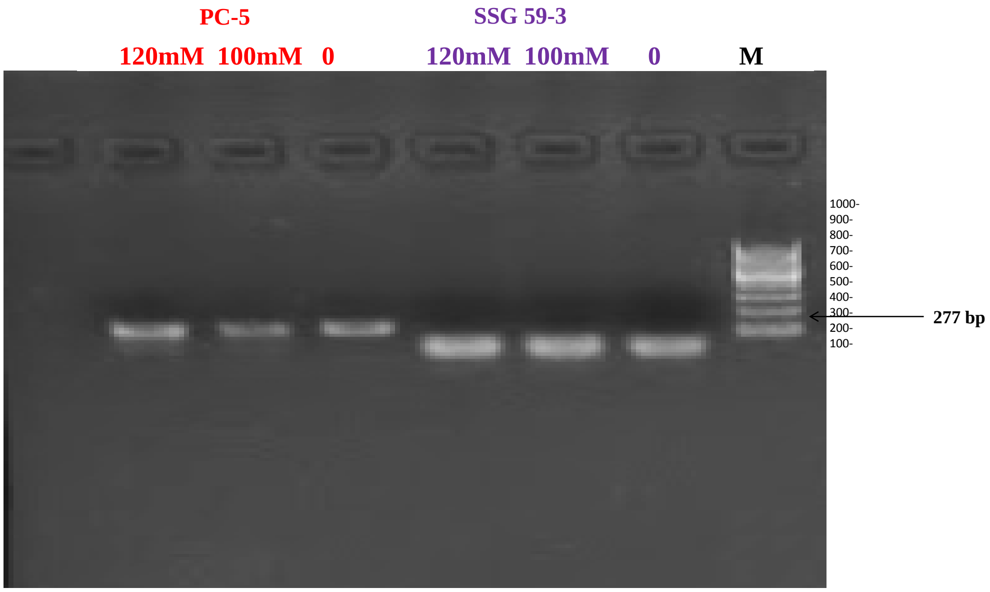
PC-5
120mM 100mM 0
SSG 59-3
120mM 100mM 0
M
1000-
900-
800-
700-
600-
500-
400-
300-
200-
100-
277 bp

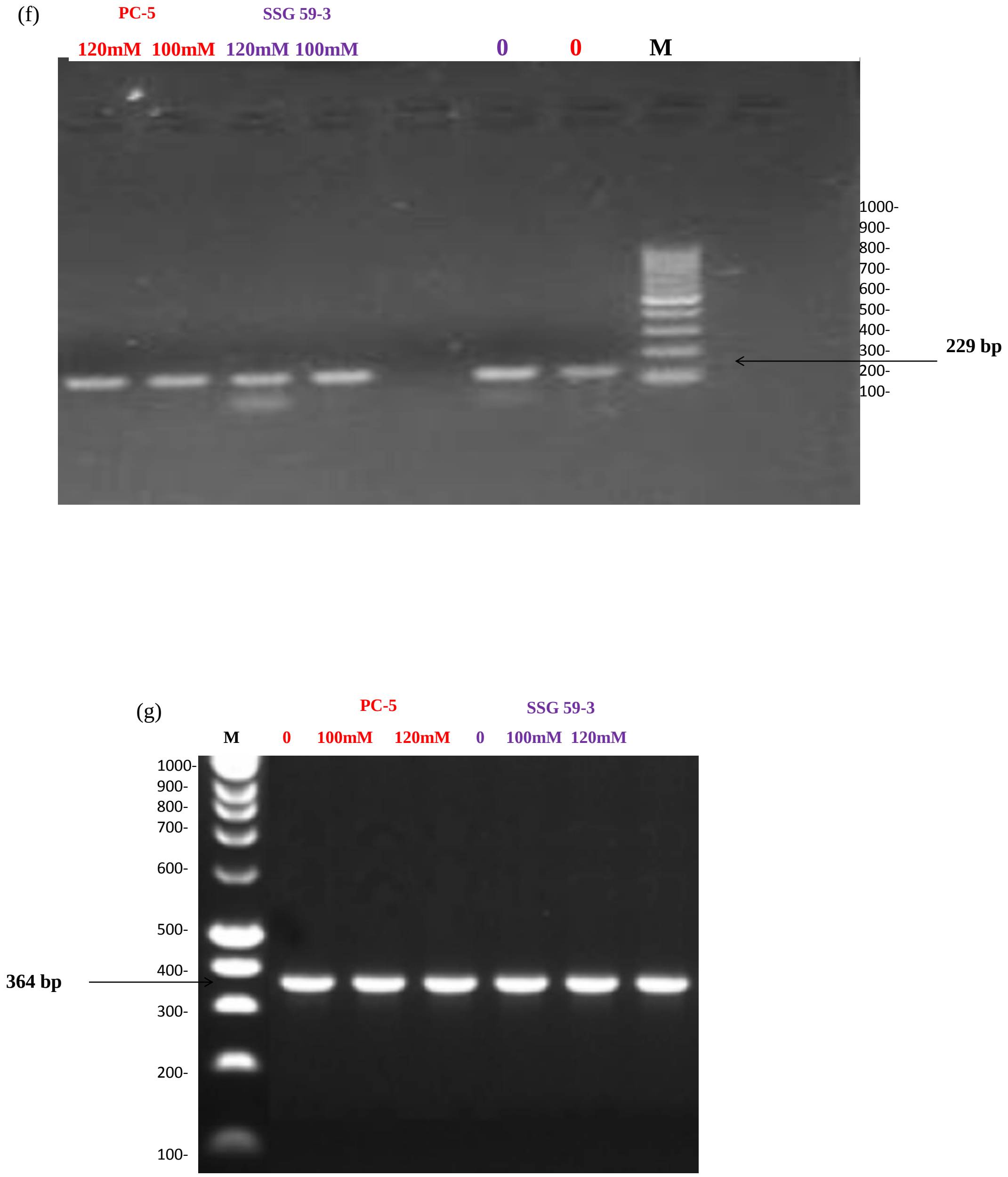

Figure S8. Semi-quantitative RT-PCR analysis of (a) Sucrose synthase (*SS*), (b) granule-bound starch synthase (*GBSS*),(c) cysteine protease (*XCP1*), (d) α-amylase synthesis (*α-amy*), (e) sodium proton antiporter (*NHX-1*), (f) vacuolar proton pyrophosphatase (*VPPase-I*), (g) actin (*Act-1*) and their relative quantification using densitometry, M: molecular marker.
